# Pre- and Postoperative Cell-Free Fetal DNA Analyses for Detecting Aneuploidy in Early Pregnancy Loss: Single-Center Prospective Cohort Study

**DOI:** 10.3390/genes16060681

**Published:** 2025-05-30

**Authors:** Takeshi Nagao, Yuki Ito, Akari Moriyama, Chika Tei, Aikou Okamoto, Osamu Samura

**Affiliations:** Department of Obstetrics and Gynecology, Jikei University School of Medicine, 3-25-8 Nishi-Shimbashi, Minato-ku, Tokyo 105-8461, Japan; takeshi.n.900113@gmail.com (T.N.);

**Keywords:** aneuploidy, cell-free fetal DNA, early pregnancy loss, fetal fraction, miscarriage, next-generation sequencing, non-invasive prenatal testing, product of conception testing

## Abstract

**Background/Objective:** Early pregnancy loss is often caused by chromosomal abnormalities, necessitating accurate diagnostic tools. While product of conception (POC) chromosomal testing is commonly used, it can be limited by culture failure or an inability to obtain fetal tissue due to spontaneous expulsion. Cell-free fetal DNA (cff DNA) analysis provides a non-invasive alternative; however, its effectiveness in early pregnancy loss, particularly in cases where fetal components are still minimal, has not been fully established. The objective of this study was to evaluate the accuracy of pre- and postoperative cff DNA analysis for detecting fetal aneuploidy by comparing the results to those of POC chromosomal testing. **Methods:** In this single-center prospective cohort study, 50 women undergoing manual vacuum aspiration for pregnancy loss before 12 weeks of gestation were enrolled (February 2022–December 2024). Cff DNA analysis was performed on maternal blood samples collected pre- and postoperatively. The primary outcome was concordance between the cff DNA and POC results. Sensitivity, specificity, and factors affecting concordance were also assessed. **Results:** Eight participants were excluded due to unsuccessful POC culture (*n* = 3), suspected maternal tissue contamination in the POC sample (*n* = 1), mosaicism (*n* = 3), or triploidy (*n* = 1), resulting in 42 evaluable cases. Preoperative cff DNA analysis showed 88.1% concordance with POC (sensitivity 86.4% and specificity 90.0%). Postoperative analysis showed 78.6% concordance (sensitivity 72.7% and specificity 85.0%). **Conclusions:** The Cff DNA analysis of preoperative and postoperative maternal blood samples showed generally good concordance with conventional POC chromosomal testing in detecting fetal aneuploidy in early pregnancy loss.

## 1. Introduction

Early pregnancy loss, often defined as the loss of pregnancy before 12 weeks of gestation, has an estimated incidence of about 10% when considering only clinically recognized pregnancies [1]. Over 70% of these cases are attributed to chromosomal abnormalities, highlighting the importance of identifying the underlying cause, particularly in patients with recurrent pregnancy loss, for whom such information is critical for guiding future pregnancy management [2]. The chromosomal testing of product of conception (POC) has been the standard method for detecting these abnormalities [3]. Its utility, however, is limited when sampling is technically difficult or when cultures fail, often resulting in inconclusive findings [4]. Cell-free fetal DNA (cff DNA) analysis is a non-invasive method that detects placental DNA fragments circulating in the maternal bloodstream to assess fetal chromosomal status [5,6]. Widely used in prenatal screening, this method has demonstrated high effectiveness, with detection rates exceeding 90% for common aneuploidies such as trisomies 21, 18, and 13 in high-risk pregnancies [7,8,9]. In recent years, cff DNA analysis has been explored in research settings for its potential utility in cases of miscarriage and stillbirth [10]. However, few studies have focused specifically on early pregnancy loss—a period that presents both high prevalence and technical challenges—and evaluated the diagnostic accuracy of cff DNA or the factors affecting that accuracy. For instance, Colley et al. analyzed 57 cases of miscarriage before 12 weeks of gestation, but in 26.3% of those cases, the exact gestational age was unknown [11]. Similarly, Balaguer et al. included 90 cases of miscarriage before 16 weeks yet did not specify whether the blood samples for cff DNA analysis were collected before or after the expulsion of fetal tissue [12]. These limitations underscore the need for clinical studies that are based on precise clinical information in early pregnancy loss.

Therefore, in this prospective study, we aimed to evaluate the diagnostic accuracy of cff DNA analysis and identify related clinical factors by comparing it with POC chromosomal testing in cases of pregnancy loss before 12 weeks of gestation. Since some miscarriages may result in the spontaneous expulsion of fetal tissue before POC sampling can be arranged, we also assessed the performance of cff DNA testing when maternal blood was collected after fetal expulsion.

## 2. Materials and Methods

### 2.1. Ethical Considerations

This study was approved by the Institutional Review Board of a university-affiliated hospital (approval no. 33-165-10782; date of approval: 13 September 2021) and was conducted according to the tenets of the Declaration of Helsinki and the national guidelines.

### 2.2. Study Design and Participants

This prospective cohort study was conducted at a university-affiliated hospital between February 2022 and December 2024. Participants were recruited from patients attending a university-affiliated obstetric outpatient clinic, including both self-referred and referred cases. The participants were women with singleton pregnancies in whom a gestational sac was confirmed by ultrasound and subsequently diagnosed with early pregnancy loss before 12 weeks of gestation. Both asymptomatic and symptomatic patients were included. Pregnancy loss diagnosis was made either at the initial ultrasound or following serial ultrasounds in cases where viability could not be determined at the first visit. Only participants who underwent POC testing via manual vacuum aspiration (MVA) were included in this study, as this approach minimizes the likelihood of culture failure associated with spontaneously expelled tissue and enables the accurate control of the timing of maternal blood sampling for cff DNA analysis relative to the uterine evacuation procedure. At our institution, MVA is the first-choice procedure for the surgical management of early pregnancy loss. Exclusion criteria were as follows: (1) the suspicion of a molar pregnancy on ultrasound; (2) an inability to obtain POC results due to culture failure; (3) a discrepancy between fetal sex in POC testing and cff DNA analysis (i.e., female karyotype in POC and male result in cff DNA), which suggests possible maternal tissue contamination in the POC sample; (4) POC results indicating triploidy or mosaicism—conditions not reliably detectable by cff DNA testing; (5) vanishing twin or miscarriage in multiple gestations; and (6) refusal to provide informed consent.

### 2.3. Protocol

Maternal blood samples were collected preoperatively and postoperatively. The preoperative blood samples were obtained immediately before the MVA procedure, specifically at the time of intravenous line placement and prior to propofol administration. The postoperative blood samples were collected 1 h (60–65 min) after the MVA, with the samples collected from the arm opposite to that used for intravenous line placement. All samples were drawn using standard venipuncture techniques.

### 2.4. Cell-Free DNA Analysis

Cff DNA analysis was performed using VeriSeq Noninvasive Prenatal Testing Solution v2 (Illumina Inc., San Diego, CA, USA). The blood sample (10 mL) was collected in Streck CFF DNA blood collection tubes (Streck, La Vista, NE, USA) and centrifuged at 1600× *g* for 10 min. Cff DNA was extracted from 1 mL of the plasma fraction. Next-generation sequencing (NGS) libraries were prepared using end-repair/A-tailing reactions, followed by the ligation of the index adapters. Each NGS library was quantified and then normalized, pooled, and sequenced on a NextSeq 500/550 (Illumina Inc., San Diego, CA, USA) with 2 × 36 base-pair paired-end reads. Data were analyzed using VeriSeq Non-Invasive Prenatal Testing Assay Software v2 (Illumina Inc., San Diego, CA, USA). The assay software calculated fetal fraction (FF) estimates and called aneuploidies of all chromosomes and partial duplications/deletions of ≥10 megabases, in alignment with the resolution of chromosomal analysis in POC testing. For quality assurance, the assay incorporated a metric called the individualized Fetal Aneuploidy Confidence Test (iFACT), which was used to evaluate whether the sequencing coverage was adequate based on the estimated FF. This ensured that the required level of clinical sensitivity was achieved. Additionally, quality control involved reviewing factors such as the distribution of cff DNA fragment lengths and the uniformity of sequencing coverage before a final result was issued. The cff DNA analysis was conducted solely for research purposes, and the results of the cff DNA analysis were not disclosed to the participants. Therefore, no confirmatory testing or clinical follow-up was performed in cases where the results were non-concordant with POC findings.

### 2.5. Sequencing Fetal Fraction and Y-Chromosome Fetal Fraction

Sequencing fetal fraction (Seq-FF) was determined using a multivariate regression model to estimate the fetal DNA fraction. Two analytical approaches were employed: elastic net regression and reduced rank regression [13]. In both approaches, the rank was determined based on the weighted rank selection criterion applied to the laboratory-developed test dataset. Genomic regions characterized by a higher proportion of shorter cff DNA fragments or an increased fragment ratio were preferentially utilized in both elastic net regression and weighted rank selection criterion.

An approximation formula was derived from a predefined set of noninvasive prenatal genetic testing samples to establish the relationship between the Y chromosome-normalized chromosome value (NCV-Y) and Seq-FF. Using the approximation formula, NCV-Y was converted into an FF, referred to as Y-chromosome fetal fraction (Y-FF). In addition, Y-FF was calculated only for samples in which cff DNA analysis detected fragments derived from the Y chromosome.

### 2.6. POC Chromosomal Testing

POC samples for chromosomal testing were collected following MVA. The type of testing performed varied according to the Japanese health insurance system guidelines. NGS was performed for the first pregnancy loss, as insurance did not cover G-banding for these cases. Meanwhile, G-banding was performed for subsequent pregnancy losses. NGS analysis was performed using nucleic acids extracted from the chorionic villi. Nucleic acid was extracted using the NucleoSpin^®^ Tissue Kit (Takara Bio, Shiga, Japan). Following extraction, whole-genome amplification was conducted on the chromosomal DNA, and sequencing libraries were prepared using the Embgenix PGT-A Kit (RUO) (CooperSurgical, Trumbull, CT, USA) with the addition of sample-specific barcodes. The libraries were then pooled to create a mixed library. Sequencing was performed using either the MiSeq system or NextSeq 2000 system (Illumina, San Diego, CA, USA), and the obtained base sequences (read sequences) were analyzed. Chromosomal aneuploidy analysis was conducted using the Embgenix™ Analysis Software v1.0.9j (CooperSurgical, Trumbull, CT, USA). Chromosomal copy numbers were considered normal if they fell within the 1.7–2.3 range for autosomes and the X chromosome in females and the 0.7–1.3 range for the X and Y chromosome in males. The resolution of G-banding was approximately 5–10 megabases, while that of NGS was approximately 8 megabases.

### 2.7. Determining Gestational Age

Gestational age was determined according to the Japan Society of Obstetrics and Gynecology guidelines [14]. The estimated due date (EDD) was calculated based on embryo transfer or ovulation date. In the absence of such data, the EDD was determined based on the last menstrual period and the individual’s menstrual cycle. If the crown-rump length (CRL) differed by more than 1 week from the calculated EDD, the EDD was recalculated based on CRL measurements. Pregnancy was considered to correspond to 5 weeks of gestation when only a gestational sac was visible.

### 2.8. Outcomes of Interest

The primary outcome of interest was the concordance rate of preoperative circulating cff DNA analysis findings with POC testing findings. Diagnostic performance was evaluated according to sensitivity (i.e., the proportion of aneuploid cases correctly identified by cff DNA analysis) and specificity (i.e., the proportion of correctly identified euploid cases). The secondary outcomes of interest were as follows: concordance between postoperative circulating cff DNA analysis findings and POC results; diagnostic performance of postoperative cff DNA analysis as evaluated according to sensitivity and specificity; and differences in maternal background and pregnancy loss-related factors between the concordant and non-concordant groups.

### 2.9. Statistical Analysis

A formal power analysis was not conducted, as this was an exploratory study designed to evaluate feasibility and preliminary diagnostic performance. The baseline participant characteristics are summarized using descriptive statistics. Categorical data are presented as frequencies and proportions and continuous data are reported as medians and ranges. Concordance rates and the sensitivity and specificity of preoperative and postoperative cffDNA analyses compared to POC testing were calculated without statistical analysis. Background characteristics were compared between the concordant and non-concordant groups using the Chi-square test for categorical variables. However, when ≥20% or more of the cells had expected counts of <5, Fisher’s exact test was applied. The Mann–Whitney U test was used for continuous variables. All subgroup analyses and statistical comparisons were performed using Fisher’s exact test. Statistical significance was defined as a two-sided *p*-value of less than 0.05. All statistical analyses were conducted using SAS version 9.4 (SAS Institute Inc., Cary, NC, USA).

## 3. Results

### 3.1. Participant Characteristics

**Figure 1:**
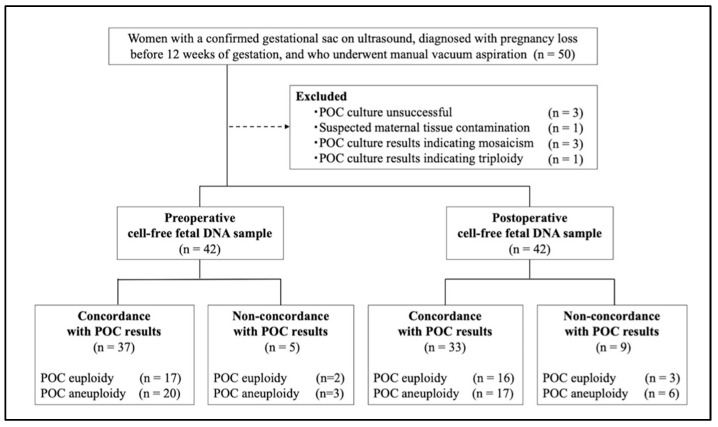
The participant selection flowchart. Women who experienced pregnancy loss before 12 weeks of gestation and were scheduled for manual vacuum aspiration are enrolled. Maternal blood samples are collected preoperatively and postoperatively for cff DNA analysis, and the results are evaluated for concordance with the POC chromosomal testing results.

Among the 50 participants enrolled, 8 participants (16.0%) were excluded due to failed POC culture (*n* = 3), suspected maternal tissue contamination indicated by a female karyotype in the POC sample and a male result in the cff DNA analysis (*n* = 1), mosaicism (*n* = 3), or triploidy (*n* = 1). Consequently, 42 participants were included in the analysis (Figure 1). The median maternal age was 39.0 years (range, 29–45 years), and the median body mass index was 21.6 kg/m^2^ (range, 17.1–27.1 kg/m^2^). Overall, 26 (61.9%) participants were nulliparous, and 19 (45.2%) participants conceived via in vitro fertilization. Regarding obstetric history, 13 (31.0%) participants had experienced their first pregnancy loss, 15 (35.7%) participants had experienced a previous pregnancy loss, 10 (23.8%) participants had experienced two pregnancy losses, and 4 (9.5%) participants had experienced ≥three pregnancy losses. The median gestational age at the estimated time of fetal demise was 59.5 days (range, 42–81 days), and the median interval from fetal demise to sample collection was 11.5 days (range, 1–43 days). Pregnancy loss occurred in 35 (83.3%) participants after ultrasound confirmation of a fetal pole, with or without cardiac activity. The remaining seven participants (16.7%) were diagnosed with pregnancy loss at the stage when only the gestational sac was visualized. The median FF rate was 7.3% (range 1.8–15.0%) in preoperative samples and 6.7% (range 1.5–29.3%) in postoperative samples. Of the 42 POC samples, 20 and 22 were euploid and aneuploid, respectively. Among the twenty-two cases of aneuploidy, there were two cases of trisomy 22; seven cases of trisomy 21; one case of trisomy 20; two cases of trisomy 16; six cases of trisomy 15; two cases of trisomy 14; one case of trisomy 13; and one case of monosomy X.

### 3.2. Primary Outcome of Interest

#### Preoperative Cff DNA Analysis

The concordance rate for the preoperative samples was 88.1% (37/42). Among the five cases with non-concordance, two had euploid and three had aneuploid results on POC testing. The sensitivity and specificity were 86.4% and 90.0%, respectively (Table 1).

### 3.3. Secondary Outcomes of Interest

#### Postoperative Cff DNA Analysis

The concordance rate in the postoperative samples was 78.6% (33/42). Among the nine cases with non-concordance, three had euploid and six had aneuploid results on POC testing. The sensitivity and specificity were 72.7% and 85.0%, respectively (Table 1).

### 3.4. Comparison of Participant Characteristics

Baseline characteristics of the concordant and non-concordant groups are summarized in Table 2. No significant differences were observed between the groups in either the preoperative or postoperative analyses. In particular, FF levels did not differ significantly between concordant and non-concordant cases in both analyses.

The Cff DNA analysis and POC analysis results were non-concordant in either preoperative or postoperative samples in nine participants (Table 3). Five participants had non-concordant results in both preoperative and postoperative samples, whereas only the postoperative samples were non-concordant in the four participants. In Case 7, none of the maternal features associated with 22q11.21 duplication—such as congenital heart defects, developmental delay, or craniofacial anomalies—were observed [15]. Similarly, in Cases 8 and 9, there were no maternal findings suggestive of Turner syndrome.

### 3.5. Subgroup Analyses

Subgroup analyses revealed no statistically significant differences in concordance rates based on maternal BMI or IVF status. Preoperative concordance was 100.0% vs. 86.8% for BMI < 18.5 (*n* = 4) vs. ≥18.5 (*n* = 38) (*p* = 1.000) and 84.2% vs. 91.3% for IVF (*n* = 19) vs. non-IVF (*n* = 23) groups (*p* = 0.644). Postoperatively, concordance was 75.0% vs. 78.9% for BMI groups (*p* = 1.000) and 78.9% vs. 78.3% for IVF groups (*p* = 1.000). Sensitivity and specificity also showed no significant differences across these subgroups. In addition, concordance rates were compared using the method of POC analysis. For preoperative cff DNA analysis, the concordance rate was 84.6% in the NGS group (*n* = 13) and 89.7% in the G-banding group (*n* = 29) (*p* = 0.637). For postoperative analysis, the concordance rate was 69.2% in the NGS group and 82.8% in the G-banding group (*p* = 0.422).

When stratified by fetal fraction, preoperative cff DNA analysis demonstrated a significantly lower concordance rate in cases with Seq-FF < 4% (*n* = 8) than in those with Seq-FF ≥ 4% (*n* = 34) (62.5% vs. 94.1%; *p* = 0.040), with a corresponding reduction in sensitivity (25.0% vs. 100.0%; *p* = 0.003). Similarly, in the postoperative analysis, concordance was lower in the Seq-FF < 4% group (62.5% vs. 82.4%; *p* = 0.336); however, the difference was not statistically significant.

## 4. Discussion

This study demonstrated the concordance rate between cff DNA analysis and conventional POC chromosomal testing in detecting fetal aneuploidy in early pregnancy loss. Preoperative samples demonstrated relatively high concordance, suggesting that cff DNA analysis performed prior to fetal expulsion may serve as a reliable non-invasive alternative when POC testing is not feasible. Postoperative samples, while showing somewhat reduced concordance, still retained reasonable diagnostic performance. These findings suggest that cff DNA analysis offers clinical utility if POC testing is impossible—for example, when conventional karyotyping fails owing to culture failure, preoperative maternal blood samples can be stored for retrospective cff DNA analysis. Similarly, if fetal tissue is lost before sampling, prompt maternal blood collection may allow for timely cff DNA testing and the recovery of chromosomal information that would otherwise be unobtainable. Therefore, although cff DNA analysis cannot fully replace POC testing, it demonstrated the potential to provide valuable complementary information regarding the etiology of pregnancy loss in both preoperative and postoperative settings.

Although this study focused on losses before 12 weeks, previous studies encompassing pregnancy losses through the second trimester have demonstrated the high sensitivity and specificity of cff DNA analysis [10,16,17]. Hartwig et al. (in losses before 22 weeks of gestation) reported a sensitivity of 85% and specificity of 93% [18], Balaguer et al. (before 16 weeks of gestation) noted 79.4% sensitivity and 100% specificity for common aneuploidies [12], and Qiao et al. (before 14 weeks of gestation) found a test success rate of 81.8% at 6 weeks, rising to 100% from 7 weeks onward [19]. Colley et al., who also investigated pregnancy loss before 12 weeks of gestation, as in the present study, reported a sensitivity of 59% and a specificity of 90% [11]. The lower sensitivity in their study may be attributed to an earlier gestational age, as the mean gestational age in their cohort was only 7.1 weeks and the mean FF was 6% (range, 2–19%). In contrast, the median gestational age in our study was 8.5 weeks (59.5 days), and the median FF rate was 7.3% in preoperative samples and 6.7% in postoperative samples.

In our study, we also examined clinical and maternal factors that may influence the accuracy of cff DNA analysis. In addition to maternal age, BMI, the FF rate, and gestational age—factors that have been explored in previous studies—we specifically assessed the timing of blood sampling in relation to the diagnosis of pregnancy loss [20,21]. Although the non-concordant group tended to have a slightly earlier gestational age than the concordant group, this difference was not statistically significant. Colley et al. suggested that detection rates of cff DNA may be influenced by gestational age, the FF rate, and serum β-hCG levels, though their study did not include statistical comparisons [11]. In contrast, Balaguer et al. and Yaron et al. reported no significant associations between cff DNA performance and gestational age, the FF rate, maternal BMI, or gestational sac size—findings consistent with our results [12,22]. However, given the limited sample size of our study, further investigation is warranted to clarify these relationships.

In Cases 1–3, POC testing detected aneuploidy, but cff DNA analysis did not, with both preoperative and postoperative Seq-FF values below 4%, suggesting an insufficient FF. In Cases 5 and 6, the reason for non-concordance is unclear; however, one possibility is an overestimation of low FF. In Cases 1, 4, and 10, Seq-FF values were notably higher than Y-FF values. According to the method of Kim et al., the elastic net model was trained on over 25,000 Non-Invasive Prenatal Test (NIPT) samples, showing a strong correlation with fetal fraction estimates based on Y-chromosome (R = 0.932–0.938) and SNP-based methods (R = 0.921) [13]. However, its accuracy may decline with low FF, causing an overestimation [13,23]. As Y-FF data were unavailable in Cases 5 and 6, the actual FF is unknown, but it may have been lower than the reported postoperative Seq-FF values (6.30% and 6.43%), potentially contributing to the non-concordance. These findings highlight the limitation of Seq-FF and support the need for future studies to combine FF estimation methods, particularly for low FF samples. Conversely, in some cases, cff DNA detected aneuploidy while the POC results were euploid. In Case 7, despite the POC showing a normal female karyotype, the preoperative cff DNA result indicated trisomy 22, and the postoperative result revealed a segmental duplication on chromosome 22. These findings may potentially reflect placental mosaicism or maternal chromosomal variation, such as asymptomatic 22q11.2 duplications, both of which have been previously reported [24]. In Cases 8 and 9, 45,X was suggested by cff DNA analysis. However, the positive predictive value for 45,X detection via cff DNA testing has been reported to be approximately 22%, indicating a high likelihood of false-positive results [25]. Therefore, the possibility of confined placental mosaicism or maternal mosaicism for 45,X should be considered as potential explanations for these findings. It should be noted that no confirmatory testing or clinical follow-up was conducted for these discordant cases, and so the interpretations should be regarded as speculative. The observed discordance between cff DNA and POC results may be attributed to two major factors: the inherent nature of cff DNA, which consists of a mixture of maternal, placental, and fetal DNA, and the challenge of accurately assessing samples with low FF. While the former represents a fundamental limitation of the method that is difficult to overcome, the latter may be addressed through improved algorithms for estimating FF, particularly in low-FF scenarios. In the subgroup analysis stratified using the commonly applied 4% cutoff in NIPT cases, Seq-FF < 4% demonstrated a lower concordance rate. However, we did not exclude cases with Seq-FF < 4% from our analysis to maintain a significant number of evaluable cases. Future studies should aim to establish appropriate FF thresholds for early pregnancy loss cases.

One strength of this study is that both preoperative and postoperative cff DNA analyses demonstrated acceptable diagnostic performance, even in early pregnancy loss. Notably, little prior research has examined cff DNA analysis following fetal expulsion. Given the short half-life of cff DNA (~16 min), we initially expected poor performance postoperatively [26]. However, sensitivity and specificity were higher than anticipated, and FF levels remained relatively stable. These findings suggest that, even when unexpected fetal expulsion occurs before scheduled POC sampling, blood collection within one hour may still provide useful diagnostic information. In this study, including only cases that underwent MVA allowed for precise control over the timing of blood sampling following uterine evacuation, enabling an accurate assessment of cff DNA levels post expulsion. Postoperative samples were collected at a fixed time point (60–65 min post MVA); thus, the impact of sampling time on diagnostic performance could not be evaluated. Interestingly, in some cases, FF levels were higher postoperatively, possibly due to physiological changes induced by the procedure—similar to those observed after external cephalic version or laser therapy for twin–twin transfusion syndrome, both of which have been associated with transient increases in circulating cff DNA levels [27,28,29]. Whether similar accuracy can be achieved after spontaneous expulsion without intervention remains uncertain, highlighting the need for further investigation.

This study has several limitations. First, it was conducted at a single institution with a relatively small cohort. Therefore, the findings should be interpreted cautiously, especially regarding statistical robustness. Second, the study population may limit generalizability. Participants were generally of advanced maternal age and had a high rate of conception via in vitro fertilization, both known risk factors for aneuploidy. These characteristics likely reflect the setting of a university-affiliated tertiary care center and may not represent the broader population experiencing early pregnancy loss. In particular, generalizability to spontaneous losses in younger, naturally conceived pregnancies may be limited. Additionally, participants exhibited a relatively low body mass index, which is known to affect FF. However, in Japan, 22.6% of pregnant women have a pre-pregnancy BMI of ≤18.5 kg/m^2^, reflecting a population trend that is more common in some Asian countries [30]. Third, the POC testing protocol was not fully standardized due to restrictions imposed by the Japanese health insurance system. Nevertheless, the detection resolutions of the two methods were comparable, with approximately 5–10 megabases for G-banding and 8 megabases for NGS. Similarly, we confirmed that there was no statistically significant difference in concordance rates between cff DNA and POC testing when stratified by the testing method (NGS vs. G-banding). Furthermore, because NGS does not require cell culture, its use may have contributed to a lower rate of culture failure compared to studies that relied solely on G-banding [31].

## 5. Conclusions

Cff DNA analysis demonstrated concordance rates of 88.1% in preoperative samples and 78.6% in postoperative samples compared to conventional POC chromosomal testing. These findings suggest that cff DNA analysis may serve as a useful non-invasive tool for detecting fetal aneuploidy in early pregnancy loss. Although not a full substitute for POC testing, cff DNA may provide complementary information on the etiology of pregnancy loss, particularly when conventional testing is not feasible.

## Figures and Tables

**Table 1:** Concordance rate and detection performance of cff DNA analysis in comparison with POC results.

	Preoperative Cff DNA Analysis	Postoperative Cff DNA Analysis
Concordance rate, n/N	37/42 (88.1%, 95% CI 74.4–96.0%)	33/42 (78.6%, 95% CI 63.2–89.7%)
Sensitivity, n/N	19/22 (86.4%, 95% CI 65.1–97.1%)	16/22 (72.7%, 95% CI 50.0–89.3%)
Specificity, n/N	18/20 (90.0%, 95% CI 68.3–98.8%)	17/20 (85.0%, 95% CI 62.1–96.8%)

Concordance refers to matching results between cff DNA analysis and POC chromosomal testing. Sensitivity indicates the proportion of aneuploid cases correctly identified by cff DNA analysis, and specificity refers to the proportion of correctly identified euploid cases.

**Table 2:** Baseline characteristics of the concordant and non-concordant groups in pre- and postoperative cff DNA analysis.

Characteristic	Preoperative Cff DNA Analysis			Postoperative Cff DNA Analysis		
	Concordant Group (*n* = 37)	Non-Concordant Group (*n* = 5)	*p* Value *	Concordant Group (*n* = 33)	Non-Concordant Group (*n* = 9)	*p* Value *
Maternal age (years), median (range)	39.0 (29–45)	39.0 (32–41)	0.585	39.0 (29–45)	39.0 (32–43)	0.853
Pre-pregnancy BMI (kg/m^2^), median (range)	21.6 (17.1–27.1)	20.5 (18.6–25.6)	0.472	21.6 (17.1–27.1)	19.8 (17.1–25.6)	0.095
Nulliparous, n (%)	22 (59.5%)	4 (80.0%)	0.628	20 (60.6%)	6 (66.7%)	0.733
Number of previous pregnancy losses, n (%)						
0	11 (29.7%)	2 (40.0%)	1.000	9 (27.3%)	4 (44.4%)	0.419
1	13 (35.1%)	2 (40.3%)	1.000	13 (39.4%)	2 (22.2%)	0.455
2	9 (24.3%)	1 (20.0%)	1.000	7 (21.2%)	3 (33.3%)	0.629
≥3	4 (10.8%)	0	1.000	4 (12.1%)	0	0.555
In vitro fertilization, n (%)	16 (43.2%)	3 (60.0%)	0.628	15 (45.5%)	4 (44.4%)	1.000
Estimated gestational age at fetal demise (days), median (range)	61.0 (42–81)	51.0 (47–59)	0.077	62.0 (42–81)	51.0 (47–68)	0.053
Interval between fetal demise and sampling (days), median (range)	11.0 (1–43)	29.0 (3–30)	0.094	11.0 (1–43)	14.0 (3–30)	0.509
Fetal confirmation by ultrasound, n (%)	31 (83.8%)	4 (80.0%)	1.000	28 (84.8%)	7 (77.8%)	1.000
Seq-FF rate (%), median (range)	7.4 (1.8–15.0)	3.5 (2.0–12.5)	0.383	8.1 (1.5–29.3)	6.3 (2.8–11.7)	0.283
Y-FF rate(%), median (range) †	5.7 (2.4–15.5)	0.2	-	8.18 (0.6–62.1)	0.8 (0.2–1.4)	0.068

BMI: body mass index; Seq-FF: sequencing fetal fraction; Y-FF: Y-chromosome fetal fraction. * Categorical variables are analyzed using the Chi-squared test. Given that 20% or more of the cells have expected counts of <5 in the previous pregnancy loss categories, Fisher’s exact test is used. Continuous variables are compared using the Mann–Whitney U test. *p* < 0.05 is considered statistically significant. The Y-chromosome was detected in preoperative samples from 15 participants in the concordant group and 1 in the non-concordant group and in postoperative samples from 14 concordant and 2 non-concordant participants. † Y-chromosome was detected in preoperative samples from 15 participants in the concordant group and 1 in the non-concordant group, and in postoperative samples from 14 concordant and 2 non-concordant participants.

**Table 3:** Characteristics of the cases with non-concordant findings between cff DNA analysis and POC testing (*n* = 9).

Case	GA (days)	BMI (kg/m^2^)	Preoperative Cff DNA Analysis			Postoperative Cff DNA Analysis			POC Testing
			Seq-FF	Y-FF	Result	Seq-FF	Y-FF	Result	
Cases with aneuploidy detected in POC results but not in cff DNA analysis									
1	47	25.6	2.59	0.57	46,XY	3.00	0.15	46,XY	47,XY,+12, der(13;14)(q10;q10),+14
2	51	21.6	1.97	-	46,XX	2.80	-	46,XX	47,XX,+16
3	53	18.6	3.46	-	46,XX	2.84	-	46,XX	47,XX,+22
4	55	17.1	7.17	3.53	47,XY,+15	4.61	1.44	46,XY	47,XY,+15
5	51	22.0	7.40	-	47,XX,+16	6.30	-	46,XX	47,XX,+16
6	68	19.8	5.33	-	47,XX,+15	6.43	-	46,XX	47,XX,+15
Cases with euploidy detected in POC results but aneuploidy detected in cff DNA analysis									
7	49	18.8	9.60	-	47,XX,+22	11.66	-	dup(22)(q11.1q13.1), XX (23.7 Mb)	46,XX
8	59	20.5	12.51	-	45,X	11.08	-	45,X	46,XX
9	68	19.8	6.75	-	46,XX	9.79	-	45,X	46,XX

The Cff DNA results that are not concordant with the POC test results are underlined in the table. GA: gestational age; BMI: body mass index; Seq-FF: sequencing fetal fraction; Y-FF: Y-chromosome fetal fraction.

## Data Availability

The data presented in this study are available on request from the corresponding author due to institutional ethics committee regulations. Access to the data will be granted upon reasonable request and approval by the ethics committee.

## Abbreviations

The following abbreviations are used in this manuscript:

POC	Product of conception
cff DNA	Cell-free fetal DNA
MVA	Manual vacuum aspiration
iFACT	Individualized fetal aneuploidy confidence test
Seq-FF	Sequencing fetal fraction
NCV-Y	Y chromosome-normalized chromosome value
Y-FF	Y-chromosome fetal fraction
CRL	Crown-rump length
